# Mindfulness as a Moderator in the Relation Between Income and Psychological Well-Being

**DOI:** 10.3389/fpsyg.2018.01477

**Published:** 2018-08-13

**Authors:** Yoshinori Sugiura, Tomoko Sugiura

**Affiliations:** ^1^Graduate School of Integrated Arts and Sciences, Hiroshima University, Higashihiroshima, Japan; ^2^The Japan Society for the Promotion of Science, Tokyo, Japan

**Keywords:** income, life satisfaction, psychological well-being, mindfulness, moderation, individual differences

## Abstract

The relation between income and life satisfaction has been found to be weak, albeit positive (*r* = 0.10–0.20). This study introduced psychological well-being (PWB) as a dependent variable predicted by income in addition to life satisfaction. Furthermore, individual differences might determine the strength of this relation, that is, act as moderators. Thus, this study introduced mindfulness as one such possible moderator. Participants (*N* = 800, 50% women, aged 20–59 years) completed an Internet questionnaire. Of them, 734 reported income and were included in the analyses. Income had weak, yet positive, zero-order correlations with life satisfaction and PWB (*r* = 0.13 and 0.11). Hierarchical regression controlling for demographics indicated that the relation between income and PWB was moderated by mindfulness facets. Specifically, among those low in not judging or describing of experiences, PWB was positively related to income. On the other hand, those high in these mindfulness dimensions indicated higher PWB irrespective of income.

## Introduction

This study will examine the relationship between income, life satisfaction, and psychological well-being (PWB). Previous studies have reported a positive but weak relationship between income and life satisfaction (e.g., Diener and Biswas-Diener, 2002), while research on the correlation between income and PWB has been sparse. This study will address a gap in the literature by examining the relation between income and PWB. The other potential advancement is the inclusion of mindfulness as a moderator, which may lead to a more nuanced understanding of the processes linking income to life satisfaction and PWB. Inclusion of a moderator may also help to explain the weak relation between income and well-being.

Studies examining the relation between income and well-being have primarily focused on life satisfaction (Diener and Biswas-Diener, 2002; Diener et al., 2010). Many studies have reported positive but weak relations between income and life satisfaction (e.g., Diener et al., 1993; Diener and Biswas-Diener, 2002). In a review, Diener and Biswas-Diener (2002) reported that in most studies, the correlation between income and life satisfaction was around *r* = 0.10–0.20. How can we explain the positive relation between income and life satisfaction? First, money enables consumption, which increases joy and reduces negative experiences (Soto and Luhmann, 2013). More income affords a higher standard of living (Christoph, 2010) and the ability to purchase comfortable goods (Gleibs et al., 2013). Second, money enhances life satisfaction by social comparison (Christoph, 2010). Wolbring et al. (2013) found that an individual’s income relative to people around that person was predictive of life satisfaction. Finally, performance self-esteem (e.g., I am proud of my educational background) mediated the relation between income and higher psychological adjustment (summation of positive psychological attributes like calm, cheerful, content, energetic, and so on) (Gebauer et al., 2013). A few explanations have been presented to explain the weakness of the relation between income and life satisfaction. First, once basic human needs are met, income diminishes its effect on life satisfaction (Christoph, 2010; Wolbring et al., 2013). Second, the role of social comparison to enhance life satisfaction is limited (Christoph, 2010; Wolbring et al., 2013). As one’s income increases, one’s standards also rise because of parallel increases in income in surrounding people. Third, joy derived from consumption is limited because of a phenomenon known as the hedonic treadmill. It is expected that people easily become accustomed to pleasures derived from increased income (e.g., Binswanger, 2006).

Meanwhile, studies on the correlation between income and psychological (eudaimonic) well-being have been sparse. PWB is characterized by a sense that one is developing toward important values, making the most of one’s potential (Ryff and Keyes, 1995). PWB was proposed to consist of six aspects derived from philosophical and psychological literature: self-acceptance (positive attitudes toward various aspects of self), environmental mastery (mastery and competence in managing environment), positive relations with others (warm and trusting relations with others), personal growth (feeling of continuous growing and expanding), purpose in life (sense of directedness for future and meaningfulness for past and present), and autonomy (self-determining and independent of social pressures) (Ryff, 1989). While the six aspects form separate factors, Ryff and Keyes (1995) found a single second-order factor fit their data. Keyes et al. (2002) also found that the six aspects loaded on a single factor of PWB. While few studies have correlated income and PWB, Kaplan et al. (2008) was a rare exception, finding income predicted PWB both cross-sectionally and longitudinally. In addition, Kaplan et al. (2008) found income predicted all dimensions of PWB, except for positive relations, which was not included in their data. Considering these studies, it is reasonable to use the total PWB score as a dependent variable in this study, while prediction of each dimension is also of importance. In considering specific aspects of PWB, their uniqueness compared to other well-being indices may be informative. For example, PWB is related to but distinguishable from subjective (hedonic) well-being (Keyes et al., 2002). Subjective well-being refers to positive cognitive/affective appraisal of one’s life, including life satisfaction, positive affect, and reduced negative affect. Among PWB subscales, environmental mastery and self-acceptance have some overlap (correlations) with subjective well-being, while the other four (autonomy, personal growth, purpose in life, and positive relations) are more distinct from subjective well-bring (Ryff, 1989; Keyes et al., 2002). Therefore, at the subscale level of PWB, relations of income to the latter four subscales may be informative.

Income may impact PWB by providing resources for self-growth and conferring a sense of contributing to society (Christoph, 2010). One may derive PWB from being a productive person, for which income serves as an explicit marker. Some of the explanations proposed for the relation between income and life satisfaction may also hold true for PWB. Social comparison and performance self-esteem may confer self-acceptance and personal growth. As Gebauer et al. (2013) measured a wide array of positive attributes, the effect of performance self-esteem may be applicable to PWB as well.

This study will introduce a moderator of the relation between income and life satisfaction/PWB in order to enable more nuanced understanding of the relation. The moderator may also explain the weakness of the relation between income and well-being. If we find a positive relation in some people, and no or a negative relation among the others, the net of these different slopes, that is, the main effect, may be weak. Individual traits are considered important moderators according to the set-point theory of well-being. The set-point theory explains that well-being is largely determined by stable personality traits; therefore, even if external factors (e.g., life events, income) affect well-being, one’s well-being will soon return to each individual’s default value (Diener et al., 2006). In other words, an individual’s personality traits work as an internal harness to maintain his/her well-being at a certain level. Empirically demonstrated individual trait moderators of the relation between income and well-being were extrinsic/intrinsic style of work motivation (Malka and Chatman, 2003), materialism (Diener and Biswas-Diener, 2002), neuroticism (Soto and Luhmann, 2013), and religiosity (Gebauer et al., 2013). Interactions involving these moderators indicated similar configurations: among those with high materialism, high extrinsic motivation, high neuroticism, or low religiosity, positive relations between income and well-being were observed. On the other hand, among those low in materialism, low in neuroticism, high in intrinsic motivation, or high in religiosity, higher well-being irrespective of income was found. In other words, a configuration of interactions referred to as a compensatory effect (c.f., Trautwein et al., 2015) was found. For example, Soto and Luhmann (2013) found that the relation between income and life satisfaction was β = 0.20 among those high in neuroticism, while β = 0.12 among those low in neuroticism. Malka and Chatman (2003) found the corresponding relation was β= 0.16 among those high in extrinsic motivation and β = −0.39 among those high in intrinsic motivation, with those having high intrinsic motivation showing overall higher subjective well-being.

This study will introduce mindfulness as a potential moderator of the relation between income and life satisfaction/PWB. Mindfulness is defined as “paying attention in a particular way: on purpose, in the present moment, and non-judgmentally” (Kabat-Zinn, 1994, p. 4). Individual differences in mindfulness have been conceptualized to have five facets (factors): observing experiences (e.g., sensations, perceptions, thoughts, and feelings), acting with awareness (noticing what is happening in the present moment), describing or labeling experiences (finding proper words fitting experiences), not judging experiences (not criticizing one’s experiences), and being non-reactive to inner experiences (not being overwhelmed by one’s experience and staying aware of it) (Baer et al., 2006). Overall, we expect that a positive correlation between income and life satisfaction and PWB may be found under low mindfulness. The rationale for expecting moderation by specific facets is detailed below.

While income may enhance life satisfaction and PWB by social comparison (i.e., I am satisfied or feel I am developing, as I earn better than my neighbors) (Christoph, 2010; Wolbring et al., 2013), those who score high on the non-judging facet of mindfulness will be less amenable to such social comparison. Such a nature of the non-judging facet is suggested by findings that a non-judging attitude is strongly related to self-compassion (e.g., Hollis-Walker and Colosimo, 2011), which reflects a caring and non-judgmental attitude toward oneself (Neff, 2003). Self-compassion is less reliant on social comparison for feelings of self-worth compared to self-esteem (Neff and Vonk, 2009). In addition, even when people have thoughts linking income and self-acceptance or personal growth, those with high non-judging may be less affected by such thoughts. Such a nature of non-judging may be exemplified by the scale items that measure it, for example, “Usually when I have distressing thoughts or images, I judge myself as good or bad, depending on what the thought/image is about [reversed].” Consistent with this proposed function of non-judging, mindfulness was shown to reduce conditional goal-setting, which is the tendency for happiness and self-worth to be dependent on specific goals (Crane et al., 2010a,b). Finally, Brown et al. (2009) found that mindfulness was negatively correlated with financial desire discrepancy. If one is less bothered by their income, income may lose its effect on life satisfaction and PWB. In sum, among those with high non-judging attitudes, income and life satisfaction/PWB may not be related. Conversely, those with low non-judging attitudes may rely on income for life satisfaction/PWB. Among PWB facets, these effects will be especially true for self-acceptance and personal growth.

Apart from social comparison and self-esteem, another vehicle for income to enhance well-being is pleasure gained from consumption. That said, mindful individuals may derive pleasure independent of material possessions. Brown and Kasser (2005) found that ecologically responsible behavior, which is less dependent on material consumption, and subjective well-being were positively related. Mindfulness explained part of this positive relationship. Mindfulness may enable one to derive pleasure from even mundane experiences. In an experiment by Levy et al. (2001), participants instructed to find new aspects of objects they did not like (inducing a state similar to that of mindfulness) had increased preference for those objects afterward. Even the act of washing dishes can bring positive affect with mindful attitudes (Hanley et al., 2015). In their study, an instruction to wash mindfully lead to increased curiosity. These processes suggest that mindful observation will allow pleasure to be derived from such experiences without much spending. Therefore, low income may not necessarily lead to lower life satisfaction and PWB for those with high mindfulness. On the other hand, those low in mindfulness may rely on income to obtain life satisfaction and PWB through consumption. As increased noticing and awareness may be a primary vehicle for this mechanism, moderation effects may be pronounced for acting with awareness and observing of experiences facets, which capture awareness and noticing of ongoing experiences (e.g., “I rush through activities without being really attentive to them [reversed]” for acting with awareness; “When I take a shower or a bath, I stay alert to the sensations of water on my body” for observing). Acting with awareness and observing will enhance enjoyment of experiences, thus enabling one to derive pleasure from every aspect of daily experiences even with less spending. The above explanations suggest that mindfulness will have more pervasive effects on life satisfaction/PWB, not limited to the moderation of the effect of income. For example, mindfulness was shown to be correlated with PWB (Hollis-Walker and Colosimo, 2011). Based on this reasoning, we also expected a main effect of mindfulness on life satisfaction and PWB.

We hypothesized that mindfulness would moderate the relation between income and well-being. Under low mindfulness, a positive relation between income and life satisfaction and PWB will be observed as such individuals are prone to being affected by beliefs linking income and life satisfaction/PWB and will not be able to gain enjoyment without spending. More specifically, non-judging attitudes, acting with awareness, and observing of experiences may be most potent as moderators. On the other hand, high mindfulness may be associated with higher life satisfaction and PWB, irrespective of income. Therefore, a compensatory interaction was predicted. We also predicted a main effect of mindfulness on life satisfaction and PWB.

## Materials and Methods

### Participants

Participants were sourced from the registered participant pool of a web-based survey company (*N* = 800, 50% women). The mean age was 39.99 years (*SD* 10.62; range 20–59). A summary of demographic variables is reported in **Table 1**. The original sample size (*N* = 800) had statistical power of 99% (two-tailed) in detecting correlation of *r* = 0.15 (based on reported income–life satisfaction correlations in the review by Diener and Biswas-Diener, 2002). After excluding those who did not report their income (*n* = 66), the corresponding estimate remained at 98%.

**Table 1:** Participants’ demographic distribution (%, total *N* = 734).

**Sex**	
Male	50.41%
Female	49.59%
**Marital status**	
Not married	45.37%
Married	54.63%
**Level of education**	
Junior high school	2.21%
High school	23.77%
Vocational school	13.58%
**Junior college**	11.72%
College	40.92%
Graduate school	7.13%
Other	0.68%
**Employment status**	
Company employee (ordinary employee)	26.32%
Company employee (manager)	8.49%
Company management (owner or officer)	2.38%
Civil servant, teacher/school employee, or non-profit employee	8.15%
Temporary or contract worker	6.96%
Self-employed (commerce, industry, or service)	5.77%
agricultural, forestry, or fishery worker	0.17%
Professional (attorney, licensed tax accountant, or other law Professional)	0.34%
Professional (physician or other health care professional)	2.55%
Part-time worker	14.26%
Other type of worker	2.72%
Full-time homemaker	14.94%
Unemployed	6.96%

*Figures do not sum to exactly 100 because of rounding.*

### Procedure

Participants from the registered participant pool of a web-based survey company were asked to complete an Internet questionnaire survey. We included participants from a wide age range, grouped into four levels (20–29, 30–39, 40–49, and 50–59 years), and an equal number of male and female participants. Therefore, eight subgroups (*n* = 100) were predetermined based on age and sex. Within each group, participants were randomly selected and sent an email inviting them to take part in the study. This procedure was continued until each subgroup had 100 participants. Researchers were blind to who was selected within each stratified group and to the identity of selected participants.

This study was carried out in accordance with the recommendations of the Ethical Review Board of Hiroshima University Graduate School of Integrated Arts and Sciences. The protocol was approved by the Ethical Review Board of Hiroshima University Graduate School of Integrated Arts and Sciences. All subjects gave written informed consent in accordance with the Declaration of Helsinki. Before beginning, the nature and purpose of the study were explained to participants, and they were told that they were free to refuse to participate. They were also told that data would be treated and analyzed anonymously, and confidentiality was strictly preserved with the web-based survey system. Participants were asked to complete the questionnaires only if they agreed to participate in the study, which was done by selecting a radio button that served as electronic written informed consent. Specifically, the explanation to participants said the following: (a) Participation is voluntary; (b) The survey intended to examine factors affecting happiness; (c) Some questions may be related to meditation or psychotherapies. Some people may find some of the questions to be delicate matters; (d) The results are used only for research purposes and treated statistically, that is, no analysis targeted at specific individuals will be conducted; and (e) Data are treated strictly confidentially. The raw data supporting the conclusions of this manuscript will be made available by the authors, without undue reservation, to any qualified researcher.

### Measures

For full questionnaire items, see Appendix 1 in **Supplementary Material**.

#### Income

**FIGURE 1:**
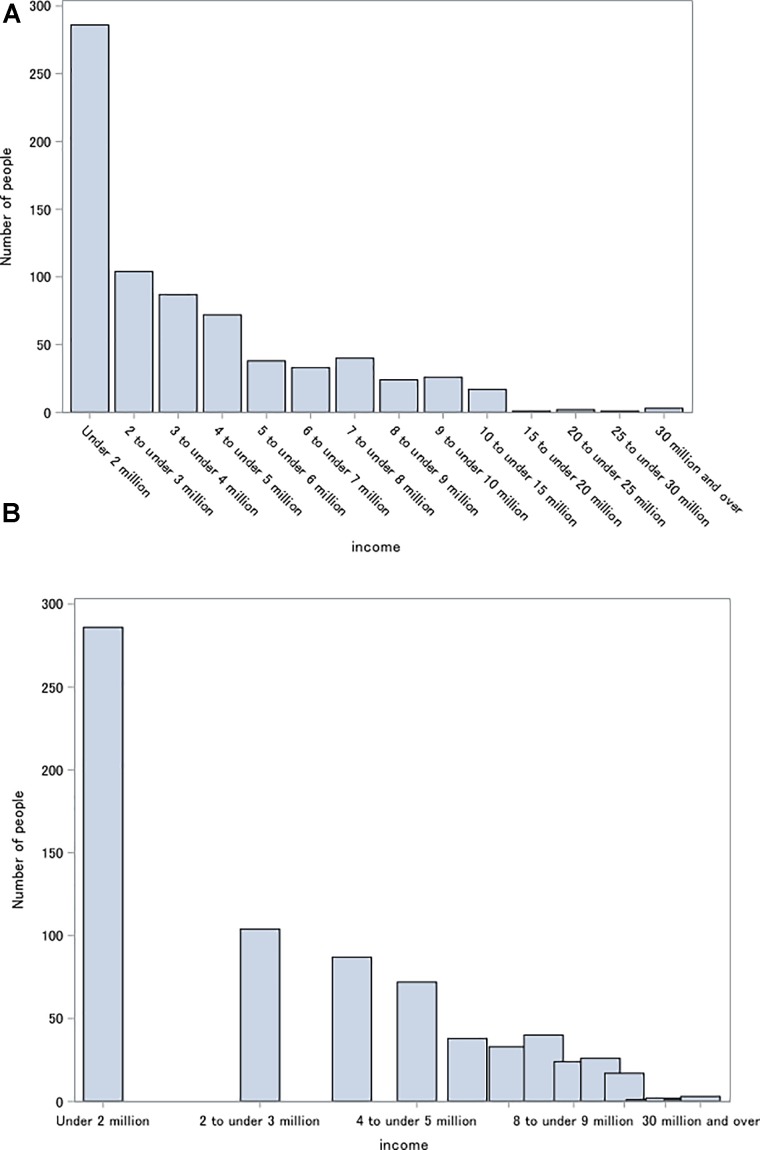
Distribution of income (Yen). Of 800 participants, 734 reported income. They reported their income by selecting from among 14 categories of ranges depicted under each bar in this graph. **(A)** Raw data. **(B)** Log transformed.

Individual income per year was rated across the 14 ranges shown in **Figure 1** divided by one million Japanese Yen (US $1 = approximately ¥114 as of February of 2017). One additional option was also included to allow participants to not provide their income if they chose.

#### Five Facet Mindfulness Questionnaire (FFMQ; Baer et al., 2006)

The FFMQ includes 39 items, each of which is rated on a five-point Likert scale that ranges from 1 (*never or very rarely true*) to 5 (*very often or always true*) and is used to measure individual differences in mindfulness. This instrument was derived using a joint factor analysis of existing mindfulness measures and comprises the following five factors: observing experiences (e.g., When I take a shower or a bath, I stay alert to the sensations of water on my body), acting with awareness (e.g., I am easily distracted [reversed]), describing or labeling experiences (e.g., I can usually describe how I feel at the moment in considerable detail), not judging experiences (e.g., Usually when I have distressing thoughts or images, I judge myself as good or bad, depending what the thought/image is about [reversed]), and being non-reactive to inner experiences (e.g., I watch my feelings without getting lost in them). Sugiura et al. (2012) constructed the Japanese version and confirmed that it had comparable psychometric properties to the original version. Specifically, the factor structure was replicated, the total score and subscales had acceptable to good internal consistency, and the mindfulness facets were positively correlated with adaptive psychological traits, indicating acceptable construct validity.

#### Satisfaction With Life Scale (SWLS; Diener et al., 1985)

The SWLS is a widely used measure of life satisfaction comprising five items rated on a 7-point Likert scale ranging from 1 (*strongly disagree*) to 7 (*strongly agree*) to capture one’s overall evaluations of one’s life. It has strong psychometric properties (Pavot and Diener, 1993). The Japanese version developed by Suh et al. (1998) with back-translation was used in this study.

#### Psychological Well-Being (PWB) Scale (Nishida, 2000)

The PWB scale has six subscales: Self-Acceptance, Environmental Mastery, Positive Relations with Others, Personal Growth, Purpose in Life, and Autonomy. Nishida (2000) developed a Japanese version of the PWB scale (not a direct translation) based on Ryff (1989) original scale. Forty-three items were rated on a 6-point scale from 1 (*strongly disagree*) to 6 (*strongly agree*). Nishida (2000) found a similar set of six factors among 241 Japanese women (ages 25–63). The Japanese version showed a six-factor structure under one superordinate factor in 232 students (Sugiura, personal communication, May 2, 2016), consistent with the original factor structure (Ryff and Keyes, 1995). The total score demonstrated excellent internal consistency in this study (Cronbach’s α = 0.94). The PWB scale also has expected relationships with existing well-being measures (Nishida, 2000). The SWLS and PWB scale were positively correlated in the present sample (*r* = 0.60; *p* < 0.001), consistent with previous findings (Keyes et al., 2002).

### Statistical Analyses

Scores for psychological measures were calculated by summing item ratings. Log transformation was conducted on income to be consistent with most previous studies correlating income and life satisfaction (e.g., Kahneman and Deaton, 2010) and PWB (Kaplan et al., 2008).

Hierarchical regression analyses predicting life satisfaction and PWB were conducted. Socio-demographic variables were dummy coded and entered in Step 1. They consisted of age, sex, residence (prefecture), job, education, and marital status (see **Table 1**). Then, main effects of the five mindfulness facets and log-transformed income were entered in Step 2 followed by their interactions (income × mindfulness facets) in Step 3. Statistical significance was set at the conventional 5% (two-tailed) throughout the paper. Mindfulness scores were mean-centered to prevent multicollinearity.

Analyses were conducted with SAS 9.4 and free statistical software R 3.4.0. Regression analyses were conducted with R lm package in QuantPsyc (Fletcher, 2012). When an interaction was significant, simple slopes were probed with an online tool by Preacher et al. (2004) and Process macro for SPSS (Hayes, 2017). Simple slopes analysis was conducted for *M* ± 1 *SD* of the relevant mindfulness facets.

We conducted *post hoc* analyses to further elucidate the interactions. To evaluate the magnitudes of interactions, *post hoc* regressions were conducted predicting life satisfaction or PWB only by income and one of the significant moderators as predictors. All variables were standardized prior to these regression analyses, thus enabling simple slope coefficients to be interpreted in the same scale as correlation coefficients. In addition, we ran regressions predicting the six subscales of PWB, with a similar process to those done for total scores. However, to reduce Type 1 error due to repeated analyses, Bonferroni’s correction was applied, setting statistical significance at *p* < 0.0083.

## Results

### Descriptive Statistics and Correlations

Sixty-six participants (9.9%) opted not to provide income data; therefore, these participants were excluded from the subsequent analyses, leaving *n* = 734. Income distribution is displayed in **Figure 1**, both before and after log transformation. Log-transformed income was used for analyses to be consistent with convention in this field (e.g., Kaplan et al., 2008; Kahneman and Deaton, 2010).

Descriptive statistics for income and the psychological measures are reported in **Table 2**, with all subscales exhibiting good to excellent alpha reliability (α = 0.80–0.94). **Table 3** displays the correlations among variables. Zero-order correlations between life satisfaction/PWB and income were computed to examine the effect of log transformation on their relationships. Raw and log income were correlated with two well-being indices to an almost identical degree (*r* = 0.13, *p* < 0.001 with life satisfaction; *r* = 0.11, *p* < 0.01 with PWB) (**Table 3**). Therefore, results with log income are reported to be consistent with the convention in this field.

**Table 2:** Descriptive statistics and internal consistency of income and psychological variables.

	*M*	*SD*	Skewness	Kurtosis	Min^a^	Max^a^	a
Income	3.27	2.72	1.26	0.94	1.00	14.00	–
Income (log)	0.86	0.80	0.31	−1.29	0.00	2.64	–
Observing	22.62	5.29	0.00	0.44	8.00	40.00	0.82
Describing	23.13	5.12	−0.08	1.11	8.00	40.00	0.81
Acting with awareness	27.48	5.28	0.00	0.07	10.00	40.00	0.85
Non-judging	25.77	5.27	0.07	0.63	8.00	40.00	0.84
Non-reacting	19.89	4.40	−0.01	1.07	7.00	35.00	0.80
SWLS	18.76	6.21	−0.20	−0.14	5.00	35.00	0.88
PWBS	162.92	26.49	0.27	0.50	82.00	246.00	0.94

*SWLS, Satisfaction with Life Scale; PWBS, Psychological Well-Being Scale. N = 734 (those who did not report their income were omitted). ^a^Measured values.*

**Table 3:** Correlations among income and psychological variables.

	Income (log)	Observing	Describing	Acting with Awareness	Non-judging	Non-reacting	SWLS	PWBS
Income (raw)	0.95^∗∗∗^	−0.10^∗∗∗^	0.11^∗∗^	0.04	−0.01	0.11^∗∗^	0.13^∗∗∗^	0.11^∗∗^
Income (log)	1.00	−0.10	0.12	0.03	−0.02	0.11^∗∗^	0.13^∗∗∗^	0.11^∗∗^
Observing		1.00	29^∗∗∗^	−0.25^∗∗∗^	−0.55^∗∗∗^	0.53^∗∗∗^	0.16^∗∗∗^	0.26^∗∗∗^
Describing			1.00	0.31^∗∗∗^	−0.06	44^∗∗∗^	0.35^∗∗∗^	0.52^∗∗∗^
Acting with awareness				1.00	0.51^∗∗∗^	−0.07	0.10^∗∗^	0.35^∗∗∗^
Non-judging					1.00	−0.31^∗∗∗^	0.09^∗^	0.09^∗^
Non-reacting						1.00	0.32^∗∗∗^	0.41^∗∗∗^
SWLS							1.00	0.60^∗∗∗^
PWBS								1.00

*SWLS, Satisfaction with Life Scale; PWBS, Psychological Well-Being Scale. N = 734 (those who did not report their income were omitted). ^∗^p < 0.05; ^∗∗^p < 0.01; ^∗∗∗^p < 0.001.*

### Moderation

Hierarchical regression analyses were conducted. Tolerance scores for income and psychological factors, including their interactions ranged from 0.21 to 0.34, suggesting that multicollinearity was not of concern in this data. In predicting life satisfaction (**Table 4**), Step 1 was significant (*R*^2^ = 0.18, *p* = 0.0000). Increment in Step 2 was also significant (Δ*R*^2^ = 0.16, *p* = 0.0000). Among Step 2 predictors, income did not predict well-being (*B* = 0.54; *p* = 0.2064). On the other hand, mindfulness facets had positive regression coefficients on life satisfaction (*B*s = 0.16–0.34; *p*s = 0.0000–0.0306), except for acting with awareness (*B* = 0.05; *p* = 0.4510). Step 3 (interactions) was not significant overall (Δ*R*^2^ = 0.01, *p* = 0.1420).

**Table 4:** Hierarchical multiple regression analyses predicting life satisfaction by income and mindfulness.

Predictor	Δ*R*^2^	*p*	*B*	*p*	95% CI
Step 1	0.18	0.0000			
Control variables^a^					
Step 2	0.16	0.0000			
Income (log)			0.54	0.2064	[−0.30, 1.38]
Observing			0.16	0.0306	[0.02, 0.31]
Describing			0.21	0.0028	[0.07, 0.34]
Acting with awareness			0.05	0.4510	[−0.09, 0.20]
Non-judging			0.30	0.0000	[0.16, 0.44]
Non-reacting			0.34	0.0001	[0.17, 0.51]
Step 3	0.01	0.1420			
Income × Observing			−0.05	0.4278	[−0.19, 0.08]
Income × Describing			0.06	0.3071	[−0.06, 0.18]
Income × Acting with Awareness			−0.18	0.0050	[−0.30, −0.05]
Income × Non-judging			0.05	0.4909	[−0.09, 0.18]
Income × Non-reacting			0.01	0.8697	[−0.13, 0.15]

*^a^Age, sex, residence (prefecture), job, education, and marital status (dummy coded).*

In predicting PWB (**Table 5**), Step 1 was significant (*R*^2^ = 0.16, *p* = 0.0001). The increment in Step 2 was also significant (Δ*R*^2^ = 0.33, *p* = 0.0000). Among Step 2 predictors, income did not predict PWB (*B* = 1.87; *p* = 0.24). On the other hand, all mindfulness facets had positive regression coefficients on PWB (*B*s = 0.78–1.82; *p* = 0.000–0.001). The increment in Step 3 (interactions) was significant overall (Δ*R*^2^ = 0.17, *p* = 0.0128). More specifically, income × non-judging (*B* = −0.68; *p* = 0.0077) and income × describing (*B* = −0.52; *p* = 0.0222) achieved significance.

**Table 5:** Hierarchical multiple regression analyses predicting psychological well-being by income and mindfulness.

Predictor	Δ*R*^2^	*p*	*B*	*p*	95% CI
Step 1	0.16	0.0001			
Control variables^a^					
Step 2	0.33	0.0000			
Income (log)			1.87	0.2447	[−1.28, 5.01]
Observing			1.47	0.0000	[0.92, 2.02]
Describing			1.82	0.0000	[1.31, 2.32]
Acting with awareness			0.78	0.0040	[0.25, 1.31]
Non-judging			1.39	0.0000	[0.86, 1.92]
Non-reacting			1.06	0.0011	[0.42, 1.69]
Step 3	0.17	0.0128			
Income × Observing			−0.48	0.0552	[−0.98, 0.01]
Income × Describing			−0.52	0.0222	[−0.96, −0.07]
Income × Acting with Awareness			0.45	0.0585	[−0.02, 0.91]
Income × Non-judging			−0.68	0.0077	[−1.18, −0.18]
Income × Non-reacting			0.32	0.2441	[−0.22, 0.85]

*^a^Age, sex, residence (prefecture), job, education, and marital status (dummy coded).*

**FIGURE 2:**
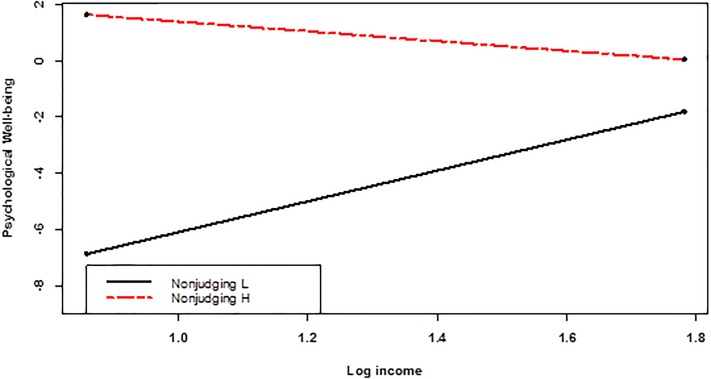
The relation between income and psychological well-being (controlling for demographics) was moderated by the non-judging facet of mindfulness.

**FIGURE 3:**
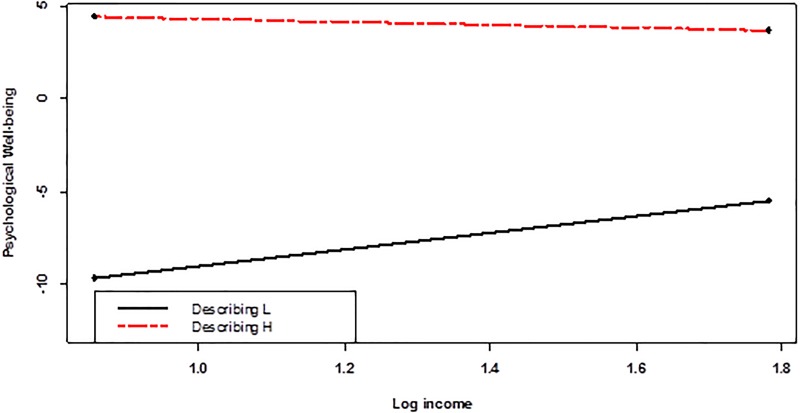
The relation between income and psychological well-being (controlling for demographics) was moderated by the describing facet of mindfulness.

As to these two mindfulness facets, simple slopes predicting PWB were probed for 1 *SD* above/below means for moderators. As **Figures 2**, **3** show, configuration of interactions were similar across two moderators, both indicating compensatory patterns. Among those low in non-judging attitudes, income was positively related to PWB (*B* = 5.46; *p* = 0.0085), while among those high in non-judging attitudes, the relation was non-significant (*B* = −1.73; *p* = 0.412) (**Figure 2**). Similarly, among those low in describing experiences, income was positively related to PWB (*B* = 4.51; *p* = 0.0259), while among those high in non-judging attitudes, the relation was non-significant (*B* = −0.78; *p* = 0.6871) (**Figure 3**). In both cases, the main effects of mindfulness facets were significant (*B* = 1.39, *p* = 0.0000 for non-judging; *B* = 1.82 and *p* = 0.0000 for describing; see **Table 5**). Therefore, as can be seen from **Figures 2**, **3**, those high in these mindfulness dimensions showed higher well-being irrespective of income.

While **Figures 2**, **3** depict clear interactions, associated simple slope coefficients are difficult to evaluate in strength, as there are many covariates including categorical ones and interactions, making standardized estimates difficult. Therefore, to evaluate the magnitudes of simple slopes, we conducted two *post hoc* regressions predicting PWB only with income and non-judging or describing. All variables were standardized prior to regression analyses. When non-judging score was low, the simple slope on PWB was β = 0.22 (*p* = 0.0000), while among those with high non-judging, the corresponding figure was β = 0.01 (*p* = 0.8559). Across the entire range of non-judging, simple slopes spanned from β = −0.17 to β = 0.47, with β > 0.08 achieved significance (*p* < 0.05). When the describing score was low, the simple slope on PWB was β = 0.12 (*p* = 0.0073), while among those with high describing, the corresponding figure was β = −0.02 (*p* = 0.7144). Across the entire range of describing, the simple slopes spanned from β = −0.18 to β = 0.26, with β > 0.06 achieved significance (*p* < 0.05).

Finally, we conducted additional regressions predicting the six psychological subscales to see specific relationships. With significance level at *p* < 0.0083, the income × acting with awareness interaction was significant for Autonomy (*B* = 0.16, *p* = 0.0022). Income × observing (*B* = −0.20, *p* = 0.0079) and income × non-judging (*B* = −0.20, *p* = 0.0073) were significant interactions for personal growth. Income was negatively correlated with autonomy when acting with awareness was low (*B* = −0.97, *p* = 0.0293), while income was positively related to personal growth when non-judging or observing were low (*B* = 1.44, *p* = 0.0176; and *B* = 1.41, *p* = 0.0166, respectively).

## Discussion

The zero-order correlation between income with life satisfaction and PWB was significantly positive but weak (*rs* = 0.13 and 0.11), corroborating many previous findings (e.g., *r* = 0.10–0.20; Diener and Biswas-Diener, 2002, for life satisfaction). Overall, mindfulness dimensions had positive main effects on well-being beyond demographic variables such that those high in mindfulness enjoyed higher life satisfaction and PWB. No main effect of income on life satisfaction and PWB was significant after controlling for demographic variables and mindfulness facets. Moderation by mindfulness facets was found only for PWB. Specifically, among those low in non-judging or describing, PWB was related to income. On the other hand, those who scored high on these mindfulness facets exhibited higher PWB irrespective of income level. That is, a compensatory interaction was found (Trautwein et al., 2015).

### Moderating Effect of Mindfulness for the Link Between Income and Psychological Well-Being

In predicting PWB, non-judgmental attitudes toward experiences and describing of experiences had moderating effects, such that income was positively related to PWB only for those who scored low in these two mindfulness facets. On the other hand, individuals scoring high in these (and other) mindfulness dimensions had higher PWB, irrespective of income.

Income may have afforded resources for self-growth for those low in mindfulness. In addition, those without non-judgmental attitudes may be affected by views such as “To be happy, I need money.” Such attitudes will make individuals rely on conspicuous external markers of well-being, such as income. Conversely, with non-judging attitudes, one can feel self-worth and a sense of continuous growing irrespective of income. Those scoring high in non-judging may also be less prone to consider high income as a prerequisite for well-being. This is possible in view of the relation between mindfulness and conditional goal-setting (Crane et al., 2010b). Non-judging attitudes may also have protected individuals from aversive social comparison. In other words, such individuals may be protected from thinking, “To be a worthy person, I have to earn more than others.” This is possible in view of the strong relation between self-compassion and non-judging (Hollis-Walker and Colosimo, 2011).

The moderating effect of describing was unexpected. This effect may be explained by findings from emotion regulation studies. Verbal processing of negative emotional stimuli was found to reduce emotional reactions to the stimuli (Hariri et al., 2003). Therefore, individuals with more describing might have been protected from distress arising from low income. In addition, writing down positive experiences has been found to enhance PWB through increased noticing of well-being in daily life (Fava and Ruini, 2003). Describing of experiences may represent a natural propensity for this process and, thus, will make one less dependent on money.

*Post hoc* regression analyses to probe the sizes of simple slopes indicated that with low non-judging (β = 0.22) or low describing (β = 0.12), the relation between income and PWB was positive but weak in magnitude. These figures are close to previously reported correlations between income and life satisfaction (Diener and Biswas-Diener, 2002). Across the range of moderator values, non-judging indicated wider change in simple slopes, suggesting that it was a more potent moderator. This may be explained by multiple possible pathways linking non-judging to the relation between income and PWB: social comparison, self-compassion, and so on.

In regressions predicting the six subscales of PWB, a similar configuration of interactions to that for the total PWB score was found for personal growth, with moderation by observing and non-judging. Personal growth is one unique aspect of PWB (Ryff, 1989; Keyes et al., 2002). In addition, when acting with awareness was low, income was negatively related to autonomy. This negative relation may reflect the fact that high income is usually associated with increased responsibility and workload (Diener and Biswas-Diener, 2002), which may diminish autonomy. Thus, acting with awareness may protect one from such an aversive effect.

The effect of income × mindfulness was found only for PWB and not for life satisfaction. This was surprising, as studies finding a positive relation between income and well-being have almost exclusively used life satisfaction as a dependent variable. The difference might have resulted from the nature of the moderators (mindfulness). For example, describing experiences was found to enhance PWB (Fava and Ruini, 2003). Therefore, it is natural that PWB is more strongly related to mindfulness than subjective well-being. In fact, in Step 2 of the hierarchical regressions, greater variance of PWB (Δ*R*^2^ = 0.33) than SWLS (Δ*R*^2^ = 0.16) was explained by mindfulness facets (income was non-significant). To extend, the fact that a moderating effect by mindfulness was found not on life satisfaction but on PWB is understandable.

While moderation by mindfulness represents a new finding, the results are also interpretable considering previous studies on trait moderators of income and life satisfaction. Previously investigated trait moderators are related to mindfulness (Brown and Kasser, 2005; Baer et al., 2006). Mindfulness is correlated negatively with extrinsic motivation, positively with intrinsic motivation (Brown and Kasser, 2005), and negatively with neuroticism (Baer et al., 2006). Furthermore, mindfulness is conceptually related to religiosity as it was derived from spiritual practices. Kashdan and Breen (2007) found that negative evaluations of unwanted internal events, a construct closely related to low mindfulness (Baer et al., 2006), were related to materialism. Mindfulness, then, is thought to be antithetical to conditions under which a positive relation between income and life satisfaction was revealed. While most of these studies were focused on life satisfaction, their implication will hold for PWB, because intrinsic motivation (Kasser and Ryan, 1996) and materialism (Kasser et al., 2014) are closely related to PWB. Therefore, one can see that mindfulness is a unifying concept when examining conditions affecting the strength of the link between income and PWB. In addition, as it is amenable to change, it can give us practical clues to protect people from aversive effects of over-reliance on income.

### Implications

Identifying moderators of the effect of income may contribute to promotion of health as these may provide a way to protect well-being from detrimental effects of relying solely on income. Although temperamental factors have been considered representative intra-individual factors that determine well-being level in set-point theory (Diener et al., 2006), mindfulness has merits over temperamental factors in that it has been conceptualized as both a trait and state, allowing it to be cultivated through intervention. Individual differences in mindfulness may serve as a reliable factor to enhance well-being. In addition, as mindfulness is amenable to change, interventions can enhance well-being and reduce reliance on money through increased mindfulness.

### Limitations and Directions for Future Research

First, although there was a large amount of variation in participants’ socioeconomic status compared to that of most psychological surveys with student samples, our sample size was relatively limited compared to the very large samples of some social science studies (e.g., World Values Survey). Second, while this study revealed that mindfulness facets had a moderating effect in the relationship between income and PWB, the combination of these facets with potential mediators, for example, social comparison and self-compassion, may lead to the development of a more complete understanding of this relationship. Third, although this study focused on life satisfaction and PWB, a broader range of indices of well-being could be considered in the future, including psychopathologies. Fourth, as this was a cross-sectional study, longitudinal and intervention studies to increase mindfulness will be important for stronger conclusions of causality. Fifth, various indices related to money should be included in the future: wealth, buying tendencies, satisfaction with income, and so on. For example, Brown et al. (2009) found that mindfulness was negatively correlated with financial desire discrepancy.

## Data Availability

The raw data supporting the conclusions of this manuscript will be made available by the authors, without undue reservation, to any qualified researcher.

## Author Contributions

YS and TS co-planned the study. YS conducted data gathering and analyses. For manuscript drafting, both contributed equally.

## Conflict of Interest Statement

The authors declare that the research was conducted in the absence of any commercial or financial relationships that could be construed as a potential conflict of interest.
