# Supplementary material for: Mindfulness as a Moderator in the Relation Between Income and Psychological Well-Being
Source: Front Psychol. 2018 Aug 13;9:1477. doi: 10.3389/fpsyg.2018.01477 (PMC6099076; doi:10.3389/fpsyg.2018.01477)
Supplement: Supplementary file 1 [file Table_1.docx]

Supplementary Material

Mindfulness as a Moderator in

The Relation Between Income and Psychological Well-Being

Yoshinori Sugiura*, Tomoko Sugiura

*** Correspondence:** Yoshinori Sugiura: ysugiura@hiroshima-u.ac.jp

1. **Supplementary Method**

Items for each scale are shown in Appendix 1.

**Appendix 1: Scale items**

A. Items of Five-Facet Mindfulness Questionnaire

Observing experiences

When I’m walking, I deliberately notice the sensations of my body moving.

When I take a shower or a bath, I stay alert to the sensations of water on my body.

I notice how foods and drinks affect my thoughts, bodily sensations, and emotions.

I pay attention to sensations, such as the wind in my hair or sun on my face.

I pay attention to sounds, such as clocks ticking, birds chirping, or cars passing.

I notice the smells and aromas of things.

I notice visual elements in art or nature, such as colors, shapes, textures, or patterns of light and shadow.

I pay attention to how my emotions affect my thoughts and behavior.

Describing

I'm good at finding the words to describe my feelings.

I can easily put my beliefs, opinions, and expectations into words.

It's hard for me to find the words to describe what I'm thinking.*

I have trouble thinking of the right words to express how I feel about things.*

When I have a sensation in my body, it's hard for me to describe it because I can't find the right words.*

Even when I'm feeling terribly upset, I can find a way to put it into words.

My natural tendency is to put my experiences into words.

I can usually describe how I feel at the moment in considerable detail.

Acting with awareness

When I do things, my mind wanders off, and I'm easily distracted.*

I don't pay attention to what I'm doing because I'm daydreaming, worrying, or otherwise distracted.*

I am easily distracted.*

I find it difficult to stay focused on what's happening in the present.*

It seems I am “running on automatic" without much awareness of what I'm doing.*

I rush through activities without being really attentive to them.*

I do jobs or tasks automatically, without being aware of what I'm doing.*

I find myself doing things without paying attention.*

Nonjudging of experience

I criticize myself for having irrational or inappropriate emotions.*

I tell myself that I shouldn’t be feeling the way I’m feeling.*

I believe some of my thoughts are abnormal or bad, and I shouldn’t think that way.*

I make judgments about whether my thoughts are good or bad.*

I tell myself I shouldn’t be thinking the way I’m thinking.*

I think some of my emotions are bad or inappropriate, and I shouldn’t feel them.*

Usually, when I have distressing thoughts or images, I judge myself as good or bad, depending what the thought/image is about.*

I disapprove of myself when I have irrational ideas.*

Nonreactivity to inner experience

I perceive my feelings and emotions without having to react to them.

I watch my feelings without getting lost in them.

Usually, when I have distressing thoughts or images, I “step back” and am aware of the thought or image without getting taken over by it.

In difficult situations, I can pause without immediately reacting.

Usually, when I have distressing thoughts or images, I feel calm soon after.

Usually, when I have distressing thoughts or images, I am able just to notice them without reacting.

Usually, when I have distressing thoughts or images, I just notice them and let them go.

*: reversed-scored items.

B. Items of Satisfaction with Life Scale

In most ways, my life is close to my ideal.

The conditions of my life are excellent.

I am satisfied with my life.

So far, I have gotten the important things I want in life.

If I could live my life over, I would change almost nothing.

C. Items of Psychological Well-being Scale

Environmental Mastery

When negative things happen to me, I can handle them fine by myself.

I can respond flexibly to problems that occur around me.

I am responsible for where I am now, which is a consequence of the past choices I made in various situations.

As I have made good choices to adapt to my surroundings, I think I am living a life that suits me.

As I have adapted well to my surroundings, I am able to live my life fully.

I can respond flexibly to my surroundings to improve my circumstances.

Self-acceptance

I am satisfied with what I have achieved so far in my life.

I can accept myself as I am, both the good and the bad.

I would like to be a different person from who I am now.*

I often feel troubled about my personality.*

I like myself.

I feel positive about myself.

I can accept my way of living and my personality just as they are.

Autonomy

I worry about what others will think about my decisions.*

I decide my actions independently.

When I decide on something important, I rely on the judgment of others.*

The way I think tends to be influenced by circumstances and others' opinions.*

When I think about how to live my life, I am easily influenced by people's opinions.*

When I determine a course of action, I first think about whether it will be socially acceptable.*

When I make a judgment, I give priority to my own values over societal approval.

I don't let social customs dictate my behavior.

Personal growth

I enjoy taking on new challenges and discovering new aspects of myself.

I believe that it is important to take on new challenges to learn about myself and my individuality and grow as a person.

I don't see the need to acquire new experiences or knowledge.*

I hope to continue to grow in various ways.

My life is a process of learning, changing, and growing.

I don't think I can do any better than I already am.*

I like having new experiences for fun.

I believe I've reached the limits of my abilities.*

Purpose in Life

I am clear on the kind of life I want to lead.

I have dreams for my future.

I have hardly any life goals and don't see my path.*

I haven't found my life purpose.*

I always have goals for my life.

I currently feel that I am wandering through life aimlessly.*

I don't know what I really want to do.*

My life is boring. Nothing arouses my interest.*

Positive relations

When I'm with others, I feel love and intimacy.

I think that maintaining close relationships requires too much effort.*

I can feel strong empathy for others.

I feel happy when I can share my time with others.

So far, I haven't built highly reliable relationships. *

I have established warm, reliable friendships.

* reversed scored items.

1. **Supplementary Discussion**

**Main Effects of Mindfulness on Well-Being**

The main effect of mindfulness on both indices of well-being is consistent with previous results (Bajaj & Pande, 2016; Chang, Huang, & Lin, 2015; Harrington, Loffredo, & Perz, 2014; Schutte & Malouff, 2011) with the exception of the lack of effect of act with awareness on life satisfaction.

The explanation for the moderation effect by nonjudging attitudes and describing also explained their main effects on well-being. For example, nonjudging was correlated to psychological well-being, mediated by self-compassion (Hollis-Walker & Colosimo, 2011). Writing down positive experience has been found to enhance psychological well-being (Fava & Ruini, 2003). In addition, the effect of other mindfulness facets can be explained in a similar way. Observing and acting with awareness of experiences, in addition to describing, will also enhance noticing of well-being amidst even mundane experiences. Nonreacting observation items seem to combine nonjudging attitudes and noticing (e.g., Usually, when I have distressing thoughts or images, I am able just to notice them without reacting; I perceive my feelings and emotions without having to react to them.)

While previous studies finding positive correlation between mindfulness and well-being used unidimensional measure of mindfulness (Chang et al., 2015), this study extended them by using a five-factored measure to find that each dimension predicted well-being (except for acting with awareness on life satisfaction). The lack of positive effect from acting with awareness to life satisfaction is difficult to interpret. However, it should be noted that acting with awareness had significant positive zero-order correlation with life satisfaction (*r* = .10, *p* < .01). Therefore, the lack of positive effect is the result of controlling for other mindfulness dimensions. Reduction in mind-wandering may partly explain the null effects of acting with awareness on life satisfaction. Lebuda, Zabelina, and Karwowski (2016) conducted a meta-analysis of 89 reported correlations between mindfulness and creativity to find that acting with awareness has weaker relation to creativity than other aspects of mindfulness (nonjudging, etc.). Unexpected ideas during mind-wandering often lead to creativity. Mind-wandering may have other benefits such as future thinking and the ability to incubate solutions following initial attempts at solving a problem (Mooneyham & Schooler, 2013). Attentional control, characterizing acting with awareness (e.g., When I do things, my mind wanders off, and I'm easily distracted [reversed]), will inhibit mind-wandering, thus reducing the chance of unexpected ideas and other benefits (e.g., enjoyment of fantasy, and so on).

**References**

Bajaj, B., & Pande, N. (2016). Mediating role of resilience in the impact of mindfulness on life satisfaction and affect as indices of subjective well-being. *Personality and Individual Differences, 93*, 63–67.

Chang, J. H., Huang, C. L., & Lin, Y. C. (2015). Mindfulness, basic psychological needs fulfillment, and well-being. *Journal of Happiness Studies, 16,* 1149–1162.

Harrington, R., Loffredo, D. A., & Perz, C. A. (2014). Dispositional mindfulness as a positive predictor of psychological well-being and the role of the private self-consciousness insight factor. *Personality and Individual Differences, 71*, 15–18.

Lebuda, I., Zabelina, D. L., & Karwowski, M. (2016). Mind full of ideas: A meta-analysis of the mindfulness-creativity link. *Personality and Individual Differences, 93*, 22–26.

Mooneyham, B. W., & Schooler, J. W. (2013). The costs and benefits of mind-wandering: A review. *Canadian Journal of Experimental Psychology/Revue Canadienne de Psychologie Experimentale, 67*, 11–18.

Schutte, N. S., & Malouff, J. M. (2011). Emotional intelligence mediates the relationship between mindfulness and subjective well-being. *Personality and Individual Differences, 50*, 1116–1119. doi: 10.1016/j.paid.2011.01.037
